# Social Variations in Perceived Parenting Styles among Norwegian Adolescents

**DOI:** 10.1007/s12187-014-9239-5

**Published:** 2014-02-28

**Authors:** Jon Ivar Elstad, Kari Stefansen

**Affiliations:** NOVA – Norwegian Social Research, Box 3223, Elisenberg, 0208 Oslo, Norway

**Keywords:** Parenting, Adolescence, Social inequality, Family economy, Norway

## Abstract

Previous research has documented the associations between parenting and parenting styles and child and adolescent outcomes. Little is known, however, about the social structuring of parenting in contemporary Nordic welfare states. A possible hypothesis is that socioeconomic variations in parenting styles in present-day Norway will be small because of material affluence, limited income inequality, and an active welfare state. This study examines social variations in parenting as perceived by Norwegian adolescents (*N* = 1362), with a focus on four parenting style dimensions: responsiveness, demandingness, neglecting, and intrusive. Responsiveness seems to capture major divisions in parenting. Adolescents in families with fewer economic resources experienced their parents as somewhat less responsive, but responsiveness was not related to parents’ education. Low parental education was on the other hand associated with perceptions of parents as neglecting and intrusive. Viewing parents as demanding did neither vary with parental education nor with family economy. Substantial variations in parenting styles persist in present-day Norway, and these variations correspond moderately with the families’ placement in the social structure. Indicators of parenting and parenting styles may be useful indicators of some aspects of child and adolescent well-being.

## Introduction

How parents raise and rear their children is related to their offspring’s lives in many ways. Following Baumrind’s seminal work on parenting and authority (Baumrind 1971, 1978), a vast number of studies have examined the associations between parenting and child and adolescent outcomes. “Good parenting” (DeVore and Ginsburg 2005), i.e. parents who are warm and stimulating, but also able to set limits, has been linked to children’s and adolescents’ emotional well-being (Chan and Koo 2011; Kiernan and Huerta 2008), self-esteem (Chan and Koo 2011), life satisfaction (Cacioppo et al. 2013), social competence (Lamborn et al. 1991), cognitive skills (Coley et al. 2011), and school adjustment (Simons-Morton and Chen 2009). Parenting seems not only associated with children’s current situation, but also to future outcomes, as supportive parenting appears to be associated with physical health (Swanson et al. 2011) and school grades (Juang and Silbereisen 2002; Kiernan and Mensah 2011). Neglectful parenting, on the other hand, has been associated with poor school performance (Dornbush et al. 1987), offspring’s overweight (Rhee et al. 2006), and drug use (Clausen 1996).

Thus, parenting which combines high levels of warmth with moderate levels of control seems associated with higher levels of well-being among children and adolescent. It has been argued that the positive effects of this type of parenting have “transcontextual validity” and are found across different “ecological niches” in terms of parents’ social position, ethnicity and family type (Steinberg et al. 1991; Steinberg 2001).

Such studies demonstrate that parenting is part of the complex processes underlying young people’s well-being. Children’s well-being implies a focus both on the present state—children’s’ *being* in the here and now—and on children’s *becoming*, i.e. their prospects and life chances (Ben-Arieh 2008; Ben-Arieh and Frones 2011). Although the causal role of parenting has been questioned (Harris 1998; Saha et al. 2010), the studies referred to above indicate that parenting and parenting styles have links to both the being and becoming aspects. It has been noticed that “interpersonal relations are a major determinant of personal (subjective) well-being in children and adolescents” (Casas 2011:570). As the family is a main arena for developing such relationships, the quality of parenting could play a significant role for the well-being of children and adolescents.

In the present study, the purpose is to analyse parenting in Norway in the 2000s. We begin by outlining the concept of parenting style, followed by a discussion of some hypotheses about social variations in parenting in the particular Norwegian context. Thereafter, we proceed to the empirical analyses. The outcomes, i.e. scales representing four parenting dimensions, are measured by adolescents’ own assessments. This approach is in line with the “new sociology of childhood” (Ben-Arieh 2008) which emphasises that children’s and young people’s own evaluations and subjective experiences are needed in order to assess their quality of life. We regard adolescents’ perceptions of their relationship with parents as significant not only because of their associations with other outcomes, but also as meaningful in themselves, since these perceptions will indicate how adolescents feel valued and respected by their parents and how they negotiate their role in family life.

After exploring the dimensions of parenting, we examine how the experience of these parenting dimensions are socially structured. A main focus is on the associations between families’ economic resources and adolescents’ perceptions of parenting, but also indicators for age, gender, family structure, parental education, and immigrant status, are employed. A causal question is raised: Does a disadvantageous family economy *bring about* deteriorated parent-adolescent relations? Finally, findings are summarised and some comments are presented about the relevance of this study for the child indicators field.

### The Concept of Parenting Style

Parenting style has been defined as “[t]he manner in which parents treat, communicate with, discipline, monitor, and support their children” (Slicker et al. 2005). A related definition is “global parenting characteristics”, i.e. characteristics of parents’ behaviours towards their offspring across a wide range of situations (Darling and Steinberg 1993). While parenting practices are tied to specific contexts, parenting styles refer to generative structures which become manifested in parents’ doings in diverse situations. According to Darling and Steinberg, a parenting style represents a constellation of attitudes towards the child that “taken together, create an emotional climate in which the parent’s behaviors are expressed” (1993: 488). While parenting practices are usually directed towards the child’s behaviours, parenting styles convey the parent’s attitudes towards the child, for instance through tone of voice, body language, inattention, or bursts of temper.

Parenting styles are thus associated with the *relational qualities* with which parents treat their offspring, which, again according to Darling and Steinberg, are essential elements of the “successful socialization of children into the dominant culture” (1993: 487). Often, specific parenting practices do not have definite, predictable effects; rather, the results will vary with the parenting style displayed by parents. The effects of particular practices will depend on the emotional environment in the family, and “(p)arenting style alters the parents’ capacity to socialize their children by changing the effectiveness of their parenting practice. From this perspective, parenting style can be thought of as a context that moderates the relationship between specific parenting practices and specific developmental outcomes” (1993: 493).

In empirical studies, parenting styles have been operationalised in different ways. A very influential typology has been proposed by Baumrind (1978), with extensions by Maccoby and Martin (1983). Factor analyses of parenting behaviours typically extract two main dimensions, usually termed responsiveness and demandingness. Responsiveness refers to the degree to which parents are accepting, supportive and receptive, i.e. their recognition of the child’s individuality and autonomy. Demandingness refers to behavioural standards and parental reinforcement, i.e. the parents’ willingness to act as socialising agents who demand compliance with norms and rules. Other labels for similar parenting dimensions are care and protection (Parker 1990), acceptance/involvement and strictness/supervision (Lamborn et al. 1991), and support and control (Clausen 1996).

The Baumrind and Maccoby/Martin typology is constructed by dividing the responsiveness and demandingness dimensions in “high” and “low” and combining them into a four-fold table (Maccoby and Martin 1983) indicating four parenting styles: Authoritative (high on both responsiveness and demandingness), authoritarian (low responsiveness, high demandingness), permissive/indulgent (high/low) and disengaged (low/low). In this model parenting styles are seen as *configurational*, i.e. as particular combinations of the responsiveness and demandingness dimensions (Morris et al. 2013). Baumrind (2013: 23) contrasts this model to an approach she defines as variable-centred: “A typological approach to parenting treats the family as a complex living system that is more than the sum of its parts, whereas a variable-centered approach to parenting reduces molar activities of family members to levels of isolated variables such as warmth, control, and cohesiveness”. A parenting type, then, is understood as a “gestalt made up of parenting practices that interact in such a way that their joint effects differs from the sum of the individual effects of their component practices”.

A problem when constructing this typology, however, is that there are no definite cut-off points between “high” and “low” responsiveness and demandingness, and the ensuing classification may be arbitrary (Chan and Koo 2011; Wen and Hui 2012). Accordingly, some researchers (e.g. Wen and Hui 2012; Leung and Lee 2012) have preferred a *dimensional* approach. In the present study, the dimensional approach is utilised in order to explore the structure of parenting in present-day Norway. This entails a focus on variations in the key aspects, or key dimensions, of parenting behaviours which underlie parenting styles (Barber and Xia 2013). In spite of their differences, it can be argued that the typological and the dimensional approaches are basically compatible, since they share the common aim of distinguishing key aspects of parenting and their effects on child well-being and development.

### Parenting Variations and the Norwegian Context

In previous research, economic hardship has been linked to poor parenting (McLeod and Shanahan 1993; Kiernan and Huerta 2008). According to the Family Stress Model (Conger et al. 1992; Conger et al. 2010), economic problems, such as unmet material needs and inability to pay bills, tend to generate emotional stress and parental conflict. Stress may lead to lack of care and harsh disciplinary methods. The complementary Investment Model (Linver et al. 2002; Bradley and Corwyn 2002) suggests that parents in poor families will have to prioritise immediate material needs and will therefore be more inattentive towards their offspring, while families with ample economic resources are more able to “invest” in their children, both in terms of time spent and in terms of economic outlays.

Our study is set in a prosperous society with an active and supportive welfare state. One could hypothesise that in such a context, the effect of economic resources on parenting will be modest. As to income inequalities, Norway is considered to be among the more egalitarian countries in the world (OECD 2011). The Norwegian economy has furthermore been relatively insulated from the recent crises in the world economy, and most sections of the population have improved their material level of living. Indeed, median household income in fixed prices rose by as much as 33 % from 2000 to 2009 (Statistics Norway 2012). Also among most Norwegian families who qualify as “poor” according to EU’s “risk of poverty” definition, i.e. equivalised household income below 60 % of the national median (EU 2008:12), access to material resources is not insignificant. A 2003 survey among such families revealed that 55 % were home-owners, 83 % went on holiday the preceding year, 81 % had a personal computer, 80 % a car, 73 % a dishwashing machine, and 63 % of the children had their own private bedroom (Skevik 2004). Although relative poverty could harm family life (Wilkinson and Pickett 2009), the material situation in today’s Norway could have weakened the link between family economy and parenting.

In addition, the Norwegian welfare state could have modified the social variations in living conditions. Public provision of welfare for children in Norway, for instance state subsidised day-care facilities available to all children aged 1–6 years old, free schooling (private schooling is rare and 98 % of Norwegian primary school children are enrolled in municipal schools), and practically free health services, relieves parents of some of the financial burdens of parenting. The welfare state which directly and indirectly conveys norms and ideals of modern parenting to all families could have had repercussions in terms of more homogeneous parenting practices.

Demographic changes may also impact on the socially structured patterns of parenting. Socioeconomic disparities are intersected by new divisions because of changing family structures (e.g. family disruption, stepfamilies and single-parent families) and the increasing proportion of immigrants in the population. A recent British study, for instance, suggests that parenting “in contemporary UK is structured primarily by family structure and not by social class” (Chan and Koo 2011). Similar developments could have occurred in Norway, and immigrant families could have introduced parenting practices which moderate the associations between parenting and socioeconomic positions.

For such reasons, the social variations in parenting found in previous research will not necessarily be found in today’s Norway. Qualitative studies (Stefansen and Aarseth 2011; Stefansen and Farstad 2010; Stefansen and Skogen 2010) of Norwegian working-class and middle-class families suggest some classed parenting practices, not dissimilar from findings in Anglo-American studies (Lareau 2003; Gillies 2006; Reay 2005), but no quantitative study of socioeconomic differences in parenting styles in Norway has been published in recent years. From Finland, however, a country which shares many welfare state characteristics with Norway, investigations of family life during the deep economic recession in the early 1990s indicated that economic hardship had negative effects on parenting quality, in line with the Family Stress Model (Leinonen et al. 2002; Solantaus et al. 2004).

### Different Viewpoints on Parenting

Research on parenting style has utilised survey answers from parents (e.g. Solantaus et al. 2004), self-reports from children and adolescents (e.g. Wen and Hui 2012; Steinberg et al. 1991; Steinberg 2001), and assessments based on observations of parental practices and parent-child interactions (Baumrind 1971, 1978, 2013). The latter approach requires more resources, but its advantage is that it corresponds to the reciprocal nature of the parent-child relation (Chen and Berdan 2006). However, as Steinberg et al. (1991) stresses, different approaches for assessing parenting style offer different windows on what goes on in the family. The perspective of the child and the parent on the parent-child relationship may differ (Steinberg 2001), and parents do not always convey their adolescents’ feelings in accurate ways (Ben-Arieh 2008). For instance, parents have been found to be more bothered by negative interactions and minor conflicts during adolescence than are teenagers (Steinberg and Steinberg 1995). Due to immaturity and the emotional ups and downs which affect young respondents, one could speculate that adolescents’ answers would be unreliable (Ritchie and Buchanan 2010), but also parents’ self-reports can be biased if coloured by wishes to be in conformity with social norms.

In recent childhood research, the importance of children’s and adolescents’ own experiences is highlighted (Jonsson and Ostberg 2010; Sandbæk 2009). Adolescents may be acute observers (Casas 2011:565), and views from the “receivers” of parenting is important since an adolescent’s perception of the relation to his/her parents is a significant aspect of this relation. Steinberg et al. (1991) contend that if a child regards her parents as, for instance, responsive and warm, this is what matters, regardless of how the parents characterise themselves or appear to outside observers. Morris et al. (2013) make a similar point regarding children’s perception of parental control, stressing that studies indicate that adolescents who perceive their parents as intrusive and negative have increased risks for negative outcomes. The present study utilises survey answers from adolescents obtained in personal interviews, in line with this understanding of the significance of how adolescents experience their relations with parents, but when interpreting the findings, it should be kept in mind that the analysed outcomes are subjective reports.

## Data, Sample, and Methods

### Data and Sample

Data come from the project *Children’s level of living—The impact of family income*, conducted at Norwegian Social Research and co-financed by Norwegian Women’s Public Health Association (NOVA 2012; Sandbæk and Pedersen 2010; Sandbæk 2013). The project’s design was inspired by the child-centered approach in current childhood research. A sample of children born 1991–1997 was drawn from the population register as of 2000 and interviewed face-to-face in 2003, 2006, and 2009. Also one parent was interviewed in each wave. The interviews were linked to data from administrative registers and anonymised in line with the regulations stipulated by the Norwegian Data Inspectorate.

The present study analyses the 2009 interviews (*N* = 1362) conducted when the child/adolescent respondents were 12–18 years old, supplemented with the 2006 interviews (1065 respondents participated both years). Because of partial non-response, the number of respondents in separate analyses will be somewhat lower. The 2003 interviews are not used since only a few of the parenting questions were asked at that time. As usual in panel studies of long duration, sample attrition was considerable (only 38 % of the initial gross sample drawn in 2000 were interviewed in 2009). Among those who were interviewed in the first wave in 2003, however, 70 % (1362 out of 1937) participated also in 2009. A main purpose of the project was to study consequences of low income for children’s well-being, and families assumed to be “poor” (i.e. less than 60 % of the median household-equivalised disposable income) were initially oversampled. Later, as the Norwegian economy developed favourably and parents’ careers evolved, many of the families improved their economic conditions, and less than one fifth of the “poor” children in 2000 were still living in a “poor” family in 2008. Because the economic divide between the two sample parts had narrowed, they are pooled in this paper, but a dummy variable indicating subsample origin has been included in the multivariate regression analyses in order to adjust for possible effects of different sample origins.

### Outcome Variables: Key Parenting Dimensions

The adolescent respondents were asked a set of questions about parental behaviours, translated from items used in Inventory of Parent and Peer Attachment (Armsden and Greenberg 1987), Parker’s Parental Bonding Instrument (Parker 1990), and Alsaker et al.’s (1991) monitoring scale. The seventeen questions had four or five response alternatives (from “Doesn’t apply at all” to “Applies perfectly”). Response rates were high (93–97 %) on the parenting items. The Appendix Table 4 lists the questions and the results of an exploratory factor analysis which applies the usual Kaiser criterion, i.e. extracts factors with initial eigenvalues above 1.00.

The factor analysis indicates that four dimensions capture much of the variations in the adolescents’ answers. The first dimension (high loadings on six items) reflects the degree to which parents are experienced as understanding, easy to communicate with, accepting, supportive and trustworthy. This dimension is clearly similar to the main component of the Baumrind typology and is therefore termed *responsiveness* in line with established terminology (e.g. Maccoby and Martin 1983). The second dimension, also frequently found in previous research, is termed *demandingness*, as its six high-loading items suggest the extent to which parents have established rules as to behaviours and manners, and monitor their offspring’s activities. The third dimension, *neglecting* (three items), suggests the degree to which adolescents experience their parents as disinterested, unhelpful and unconcerned, while the interpretation of the fourth dimension, *intrusive* (two items), is that it reflects how parents act in overly controlling and invasive ways.

Chronbach’s alpha coefficients (Appendix Table 4) were somewhat low, but acceptable (with doubts as to the intrusive dimension, however), and scales representing the four dimensions were constructed. Values 1–5 were attached to the response alternatives, added for the highloading items, and divided by the number of items; thus, the possible range of scale values is from 1 to 5. The higher scale values, the more are parents perceived as responsive, demanding, neglecting, and intrusive, respectively. The relatively high mean score on responsiveness (4.40) and demandingness (4.19), and the quite low mean values on the intrusive (2.54), and on the neglecting dimension (1.50) in particular, suggest that the adolescents in this sample often viewed their parents positively, a finding that resonates well with previous research (Steinberg 2001).

The parenting dimensions were interrelated in characteristic ways (see Appendix Table 4). Responsiveness and demandingness were positively correlated (*r* = 0.44); thus, parents perceived as responsive were also often perceived as observant of the adolescent’s activities. Responsiveness and the neglecting dimension were, on the other hand, negatively correlated (*r* = −0.44), indicating that feelings of neglect often went together with parents perceived as less responsive. Demandingness was also negatively correlated with the neglecting dimensions, although weaker (*r* = −0.26), while the intrusive dimension had comparatively low correlations with all the three other parenting dimensions.

### Independent Variables

An advantage of the present study is that detailed tax register information for several years preceding 2009 is available, practically without missing values, enabling an assessment of the family’s economic resources independent of subjective evaluations. We constructed three family economy indicators (based on figures adjusted for inflation, 2007 = 100). *Family income* is the yearly average for 2007 and 2008 of the sum of disposable (i.e. post-tax) incomes for all household members, divided by household weights (first adult = 1.0, other adults = 0.5, each child = 0.3, cf. Eurostat 2013). *Financial assets* is the household-adjusted yearly average (2007/2008) of the monetary value of the household members’ bank accounts, stocks, shares, and similar assets which easily can be converted into cash. The importance of such financial assets for families’ level of living has been demonstrated in recent studies (Elmelech 2008; Rothwell and Han 2010). The third indicator, termed *total family economy*, is a summary indicator of the family’s access to economic resources, constructed by adding the two former measurements. Since a family’s consumption opportunities and economic security will depend both on yearly income and on access to financial assets, this composite measurement is arguably a better indicator for the family’s overall economic resources than family income.

The associations between the family economy indicators and the parenting dimensions deviated from linearity, in particular ways for each of the four parenting scales. In order to reflect these patterns, the economy variables were recoded into categories as described in the tables.

Other explanatory variables include *parental education*, i.e. the parents’ average number of years in formal education (including the compulsory 9 years), obtained from Statistics Norway’s educational register, and recoded into three categories. Those who lacked educational information (<5 %) were considered as having elementary schooling. *Non-Western immigrant* (population register information, no missing values) indicates that both parents were born in a non-OECD country. *Family structure*, based on questions asked the interviewed parent, indicates with whom the adolescent was living; both biological parents, or one biological parent (usually the mother) and a step-parent, or in a single-parent family. *Parental employment*, also based on parents’ answers, indicates whether any adult family member was employed at the time of interviewing. *Parent’s self-rated health* is a three-level ordinal variable based on the interviewed parent’s answers about his/her overall self-assessed health situation. About four per cent had missing values on these questions and were assigned the sample’s modal value.

### Statistical Analyses

First, the sample distributions and the bivariate associations between the parenting dimensions and the independent variables are displayed. These bivariate analyses may be confounded, however, since several of the independent variables are correlated (e.g. Pearson’s r between education and total family economy is +0.41). Therefore, multivariate OLS regression models are used in order to show the cross-sectional relationships between parenting, as reported in 2009, and the total family economy and the other indicators of parental and family characteristics, adjusted for other covariates.

These cross-sectional analyses do not necessarily indicate *causal* effects, however. Unmeasured circumstances such as the work history of the parents, or their personal traits and social skills, could have affected both the families’ economic resources and their parenting styles. The panel structure of the data offers a way of approximating a causal estimate (Allison 1990; Allison 2009; Halaby 2004; Guo and VanWey 1999). We assume that invariant, *unmeasured* family-related circumstances have influenced the adolescents’ perceptions of their parents’ behaviours in the same way in 2006 and 2009. We also assume that permanent circumstances for which we have data, i.e. the adolescent’s gender, immigrant status, and the parents’ educational level (which was practically Stable 2006–2009), could not produce changes from 2006 to 2009 in the adolescents’ perceptions of their parents’ behaviours. Given these assumptions, the *changes* in perceived parenting from 2006 to 2009 must be due to *changes* in the adolescents’ environments from around 2006 to around 2009. Using this logic, a more unbiased estimate of the role of family economic resources can be obtained, by analysing how changes in these resources influenced changes in the perceptions of parenting.

Accordingly, the 2006 and the 2009 interviews were used for estimating *change scores* for the parenting dimensions (i.e. 2009 scale values minus 2006 scale values). Similarly, we calculated a categorical variable indicating how the total family economy changed from 2004/2005 to 2007/2008. Also other change scores were included as predictors: A variable indicating family disruption (a dummy variable showing whether married/cohabitating parents in 2006 had split up in 2009); indicators for change in parental employment (two dummy variables representing increase and decrease, respectively, in parental employment from 2006 to 2009); and a variable indicating deterioration (versus being stable or improved) from 2006 to 2009 in the interviewed parent’s self-rated health. Together, these indicators reflect developments in the environments of the adolescents which could influence their views of their parents. The models, with changes in perceived parenting as outcomes, were estimated by OLS regression. Gender, immigrant status, and parental education were not included as predictors as they are, in principle, invariant circumstances which could not lead to changes in perceived parenting. – The analyses were performed with SPSS Version 21.

## Results

The *bivariate* associations displayed in Table 1 indicate no gender differences as regards responsiveness and neglecting, but girls, more than boys, experienced their parents as demanding, while boys more than girls experienced their parents as intrusive. Parenting was experienced as less responsive and especially less demanding and less intrusive among older (age 16–18) than among younger adolescents. Those with non-Western origin viewed their parents as slightly less responsive, slightly more neglecting, but clearly more intrusive. When the adolescents lived with both parents, more responsive and demanding, but slightly less neglecting and intrusive, parental behaviours were reported. If no adults in the family worked, parents were perceived both as more neglecting and as more intrusive, and when the interviewed parent reported less good health, less responsive, less demanding, and more intrusive parenting were appeared.

**Table 1:** Sample description and bivariate associations with parenting dimensions (*N* = 1362)

Categorical explanatory variables	% of total sample	Responsiveness		Demandingness		Neglecting		Intrusive	
		Mean value	*P*-value^a^	Mean value	*P*-value	Mean value	*P*-value	Mean value	*P*-value
Boys	47.9	4.40		4.12		1.47		2.64	
Girls	52.1	4.40	0.922	4.25	0.001	1.52	0.214	2.45	0.002
Age 12–15 in 2009	59.3	4.43		4.35		1.52		2.71	
Age 16–18 in 2009	40.7	4.36	0.007	3.95	<0.001	1.46	0.093	2.29	<0.001
Native origin	85.3	4.41		4.18		1.48		2.45	
Non-Western origin	14.7	4.33	0.046	4.25	0.207	1.59	0.036	3.08	<0.001
Intact family in 2009	62.1	4.44		4.28		1.45		2.60	
Stepfamily	12.2	4.35		4.12		1.50		2.40	
Oneparent family	25.6	4.34	0.003	4.08	<0.001	1.55	0.044	2.47	0.061
At least one parent employed	88.7	4.42		4.21		1.47		2.52	
No employment	11.3	4.35	0.131	4.22	0.828	1.61	0.011	2.72	0.050
Selfrated health interviewed parent									
Very good	29.6	4.46		4.26		1.45		2.53	
Good	43.0	4.40		4.14		1.52		2.46	
Fair/poor	27.4	4.35	0.019	4.18	0.036	1.51	0.202	2.69	0.008
Parental education (average years)									
<11 years	27.5	4.39		4.21		1.59		2.80	
11 to 15 years	44.6	4.38		4.15		1.51		2.52	
15 years and more	28.0	4.44	0.174	4.22	0.247	1.39	<0.001	2.33	<0.001
Average family income 2007/2008									
<150,000 Norwegian Kroner NOK	12.8	4.41		4.15		1.58		2.68	
150–250,000 NOK	49.2	4.38		4.21		1.53		2.60	
250–350,000 NOK	26.1	4.42		4.17		1.43		2.43	
350,000 NOK +	11.8	4.44	0.522	4.16	0.614	1.42	0.019	2.44	0.029
Average financial assets 2007/2008									
<40,000 Norwegian Kroner NOK	44.3	4.37		4.17		1.57		2.69	
40–150,000 NOK	31.3	4.38		4.20		1.47		2.41	
150–350,000 NOK	13.7	4.43		4.19		1.41		2.52	
350,000 NOK +	10.7	4.54	0.004	4.22	0.851	1.37	0.002	2.39	0.001
Average Total family economy									
<170,000 Norwegian Kroner NOK	11.7	4.35		4.10		1.62		2.72	
170–300,000 NOK	41.3	4.39		4.25		1.54		2.65	
300–450,000 NOK	23.7	4.38		4.12		1.47		2.43	
450–750,000 NOK +	14.5	4.44		4.21		1.39		2.44	
750,000 NOK +	8.8	4.52	0.051	4.19	0.077	1.39	0.002	2.34	0.002

Total number of respondents in 2009; due to missing values the bivariate analyses are based on 1286–1308 respondents

^a^ *P*-values from ANOVA F-tests

The lower half of Table 1 shows that responsiveness and demandingness had negligible associations with parental education, but low parental education was clearly associated with perceptions of more neglecting and more intrusive parents. Interestingly, the associations between the parenting dimensions and family income were generally weaker than the associations with families’ financial assets and with the composite indicator total family economy. However, both higher income, more financial assets, and a better total family economy was associated with less neglecting and intrusive parents, while demandingness, on the other hand, was practically unrelated to the economy indicators. Responsiveness, which can be viewed as a major component of the variations in perceived parenting, was unrelated to family income, but responsiveness was higher in families which scored particularly high on financial assets and total family economy.

Table 2 shows *multivariate* OLS regression analyses indicating the effects of the structural indicators while controlling for the effects of the other independent variables. Responsiveness increased consistently with a better total family economy, and responsiveness was significantly higher (using the standard *p* < 0.05 criterion) in the “richest” families, compared to families lowest in the economic hierarchy. Less responsiveness was also reported by adolescents aged 16–18 (compared to younger adolescents) and by adolescents whose parent had reported poor health, but neither parental education, gender, nor non-Western immigrant background was associated with responsiveness.

**Table 2:** Multivariate OLS regression. Four parenting dimensions as reported in 2009, effects of parental education, Total family economy 2007/2008, and background factors

	Responsiveness			Demandingness			Neglecting			Intrusive		
	B	Beta	*P*-val	B	Beta	*P*-val	B	Beta	*P*-val	B	Beta	*P*-val
Parental education Ref: <11 years												
11–15 years	−0.036	−0.036	0.326	−0.042	−0.029	0.404	−0.045	−0.034	0.337	−0.107	−0.047	0.171
15 years +	0.001	0.001	0.984	0.019	0.012	0.742	−0.143	−0.098	0.010	−0.262	−0.105	0.004
Total family economy												
Ref: <170,000 NOK												
170–300,000 NOK	0.035	0.034	0.454	0.158	0.106	0.015	−0.071	−0.053	0.249	0.030	0.013	0.765
300–450,000 NOK	0.023	0.019	0.665	0.048	0.028	0.506	−0.112	−0.073	0.097	−0.061	−0.023	0.583
450–750,000 NOK	0.078	0.055	0.182	0.137	0.066	0.090	−0.168	−0.090	0.027	0.008	0.002	0.952
750,000 NOK +	0.146	0.083	0.027	0.090	0.035	0.322	−0.169	−0.073	0.049	−0.072	−0.018	0.610
Gender Ref: Boys, girls coded 1	−0.006	−0.006	0.840	0.137	0.093	<0.001	0.050	0.038	0.173	−0.170	−0.076	0.005
Age Ref: 12–15, 16–18 coded 1	−0.079	−0.077	0.006	−0.398	−0.267	<0.001	−0.045	−0.033	0.231	−0.387	−0.170	<0.001
Non-Western background	−0.058	−0.041	0.181	0.100	0.048	0.098	0.028	0.015	0.625	0.490	0.155	<0.001
Parent’s selfassessed health	−0.042	−0.063	0.031	−0.044	−0.045	0.109	−0.003	−0.003	0.915	−0.017	−0.012	0.677
Subsample origin	−0.031	−0.024	0.412	0.120	0.064	0.024	−0.013	−0.007	0.799	−0.035	−0.012	0.666
Intercept	4.679		<0.001	4.277		<0.001	1.628		<0.001	2.992		<0.001
Adjusted R^2^	0.012			0.085			0.014			0.078		
(N)	(1286)			(1307)			(1308)			(1300)		

As to demandingness, marked age and gender variations can be observed, but neither parental education, total family economy, nor non-Western background had any noteworthy associations with this parenting dimension. As to the neglecting and intrusive dimension, Table 2 shows clear associations with parental education, and more neglecting parenting was also reported in families with a relatively poor total family economy. Girls and older adolescents reported less intrusive parenting, while adolescents from non-Western families experienced more intrusive parents. However, these regression models, as indicated by the beta coefficients and the values for explained variance, only account for a very small part of the variations in perceived parenting.

If *changes* in adolescent circumstances are followed by changes in the perceptions of parenting, it is not unlikely that these circumstances have a direct *causal* effect on the outcomes. OLS regression analyses based on this logic are reported in Table 3. The intercepts in these models indicate how reporting of parenting changed, on average, as the adolescents grew 3 years older (given zero values, i.e. no change, on the explanatory variables). Overall, both responsiveness, and demandingness and intrusive in particular, declined as the adolescents matured. Apart from this, most coefficients are low and not statistically significant. Explained variance is very low, suggesting that these explanatory variables contribute only marginally to the changes in perceptions of parenting styles—note, however, the small sample sizes in the analyses of neglecting and intrusive (explained in the table’s footnote).

**Table 3:** Changes in perceived parenting 2006–2009. Effects of change in Total family economy from 2004/5 to 2007/8 and other family changes 2006–2009

	Responsiveness			Demandingness			Neglecting^a^			Intrusive^a^		
	B	Beta	*P*-val	B	Beta	*P*-val	B	Beta	*P*-val	B	Beta	*P*-val
Change total family economy												
Ref: < +50.000 NOK												
+ 50–100.000 NOK	0.019	0.018	0.599	−0.015	−0.008	0.806	−0.175	−0.097	0.078	−0.084	−0.027	0.628
+100–200.000 NOK	0.038	0.033	0.330	−0.116	−0.059	0.077	−0.057	−0.033	0.549	−0.082	−0.027	0.620
+200.000 NOK and more	0.099	0.074	0.028	0.059	0.026	0.433	−0.174	−0.079	0.140	0.080	0.021	0.697
Family disruption	−0.041	−0.025	0.490	−0.183	−0.067	0.062	−0.134	−0.049	0.392	−0.235	−0.049	0.401
Increased employment	−0.008	−0.006	0.846	0.074	0.036	0.256	−0.047	−0.022	0.661	0.616	0.168	0.001
Reduced employment	−0.033	−0.025	0.500	0.107	0.047	0.198	−0.107	−0.047	0.412	−0.060	−0.015	0.797
Parent self-rated health worse	0.032	0.029	0.351	−0.059	−0.032	0.301	−0.116	−0.067	0.188	0.037	0.012	0.811
Intercept	−0.088		<0.001	−0.191		<0.001	0.114		0.081	−0.401		<0.001
Adjusted R^2^	0.001			0.003			0.005			0.017		
(N)	(1019)			(1036)			(397)			(392)		

^a^In 2006, items included in the neglecting and intrusive scales were only asked respondents aged 13+; (N) is therefore <400

Nevertheless, after adjustment for other environmental changes and for the constant family-related factors (both measured and unmeasured), an improved total family economy from 2004/2005 to 2008/2009 was consistently associated with higher reporting of responsiveness in 2009 than in 2006. In families with the largest economic gains (+200,000 NOK and more, 13 % of the sample), the positive change in perceived responsiveness was significantly larger than the change reported in families with practically stable (or declining) economic resources. Separate analyses indicated that this pattern occurred both in families with high and low parental education (tables not shown). Thus, Table 3 indicates that a better overall family economy tends to be accompanied by perceptions of more responsive parents—this tendency, although significant in statistical terms, was not strong, however. Table 3 also shows a weak tendency that family disruption leads to less demanding parents (*B* = −0.18, *p* = 0.06), while the astonishingly large effect of increased parental employment on intrusive parenting was probably due to chance.

## Summary and Discussion

This study indicates that Norwegian adolescents’ perceptions of their parents can, to a considerable extent, be captured by four underlying dimensions: Responsiveness, demandingness, neglecting and intrusive. Variations in responsiveness seem a particularly important aspect of the differences in parenting in contemporary Norway. The structure of parenting dimensions, as reported by adolescents, are in many ways congruent with the models developed several decades ago by Baumrind (1978, 2013). Responsiveness and demandingness emerge as two major dimensions, and the two are associated with each other, but the moderate strength of the correlation (*r* = 0.44) indicates that the two dimensions represent somewhat diverging aspects.

However, the results question whether the actual variations in parenting in Norway can be adequately reflected by only these two major dimensions. Two other dimensions, named neglecting and intrusive in this study, emerged from the adolescents’ answers. Also the neglecting and intrusive dimensions distinguish to some degree between adolescents’ experiences, and future studies should explore whether such additions to the parenting dimensions are fruitful also in other social settings. While the responsiveness dimension, and also demandingness to a certain extent, represent varying degrees of positive experiences, neglecting and intrusive parenting signify unsatisfying parent-offspring relations which research also should pay attention to.

Furthermore, the analyses revealed how the experience of parenting varied according to the adolescents’ structural positions. Girls more than boys experienced their parents as demanding, while boys experienced their parents as more intrusive. This could reflect that gendered expectations continue in spite of decades with gender equality policies (Leira and Ellingsæter 2006); parents many may still think it “natural” to monitor daughters more closely than sons. Age variations were also evident. Demanding and intrusive parenting, but also responsiveness, declined in late adolescence (age 16–18). When children grow older (Steinberg and Morris 2001), most parents will reduce their monitoring and controlling behaviours, but this age-related change in parent-offspring relationships may also be experienced, by the adolescent, as a drop in responsiveness.

Also family structure was associated with perceptions of parenting, as parents in intact families were perceived as more responsive and demanding, and slightly less neglecting. Nonetheless, the effect of family structure seems modest in this Norwegian sample, compared to recent findings from Britain (Chan and Koo 2011), which could reflect that welfare benefits and opportunities for female employment in Norway enhance the life conditions for single-parent families. Moreover, it seems that non-Western adolescents face somewhat different parenting than what a typical native adolescent experiences. Non-Western parents were perceived as slightly less responsive and slightly more neglecting, and clearly more intrusive. The reason for this could be non-Western immigrant parents’ traditional cultures. However, as educational and occupational ambitions are often quite high in non-Western immigrant families (Grodem 2009), the observed pattern could well signify the parents’ determination to make their children succeed in their new country.

A main issue for this study has been to investigate the hypothesis that the Norwegian context, characterised by material affluence, high educational levels, comparatively small income inequalities, and a supportive welfare state, would tend to weaken the associations between parenting and the families’ socioeconomic position. The findings both concur, and do not concur, with this hypothesis. The family economy seemed to have no associations with demandingness and the intrusive dimensions, and was only weakly associated with neglecting, in line with the hypothesis. Perceived responsiveness was, on the other hand, related to the *total* economic resources of the family. The tentative causal analysis indicated even a *causal* link: An improved total family economy was accompanied by a positive (but modest) change in adolescents’ reporting of parental responsiveness. This pattern suggests that also in present-day Norway, a family’s placement in the economic hierarchy influences how the parent-offspring relationship evolves.

A noteworthy finding is furthermore that variations in yearly *income* had usually negligible associations with variations in parenting. Only when the family’s financial assets were taken into account and used to form a composite indicator of the family’s *total* economic situation, a distinct association between families’ economic resources and responsive parenting emerged. Wealth and financial assets may actually be a better indicator of a family’s overall economic situation than yearly income in present-day Norway. The mechanisms linking economic resources to responsiveness cannot be further explored in these data, however. Relative deprivation might lead to parental stress which the adolescents experience as unresponsive parenting; but one could also speculate that when adolescents in an affluent society come to realise that their families are relatively unsuccessful in economic terms, they react with diminished respect and more critical attitudes towards their parents.

Parental education was, on the other hand, associated neither with responsiveness nor with demandingness, but perceptions of neglecting and intrusive parenting varied clearly with parents’ education. The assumption that higher education would imply closer knowledge of modern theories of parenting and therefore be associated with more responsiveness was not supported, but the association between education and the neglecting and intrusive dimensions could nevertheless signify that parental education influences parenting. Studies from several countries (e.g. Khoury-Kassabri and Straus 2011; McLeod and Shanahan 1993) have indicated that low socioeconomic status are related to harsh disciplinary methods. By implication, one could expect that demandingness and the intrusive dimension would vary with parental education, but in the analysed sample, this seems only to be the case for intrusive parenting, but not for demandingness. It is not unlikely that norms propagated in recent decades by schools, child health services, and mass media, could have led towards a social equalisation as regards controlling and disciplinary parental practices, and the law banning corporal punishment of children (put into force in Norway in 1987) could have “pushed” in the same direction.

### Limitations

Parenting is a composite phenomenon, and parenting *goals* and parenting *practices* do not necessarily coincide with the parenting style *dimensions* analysed here. As pointed out by Darling and Steinberg, “parenting style is theoretically independent of specific socialization content” (1993). Thus, the social and socioeconomic patterns found in this study would not necessarily be replicated if other aspects of the parenting complex had been examined. Another limitation should also be mentioned: Parenting dimensions were extracted from a set of 17 questions, but the extent to which these items represent the “universe” of parenting in Norway cannot be ascertained without broader studies. Moreover, because of the sampling design and sample attrition, the adolescents analysed here cannot be considered a random sample of Norwegian adolescents born 1991–1997. However, the main purpose of this study has been to analyse the *associations* between perceived parenting and indicators of parental and family characteristics, and associations estimated from a somewhat unrepresentative sample will seldom diverge dramatically from those found in an entirely representative sample. It can also be noted that variations both in parents’ educational level and in families’ economic conditions are well represented in the analysed sample. The sample has a low-income bias—14 % of the sample were below the “poverty line” in 2008, compared to a nation-wide poverty rate of 8 % in 2006 among families with children (see Nadim and Nielsen 2009:24)—but also well-situated families are well represented, as 38 % had equivalised incomes above the 2008 national median. A clue to the effects of attrition comes from comparing the distributions in the sample interviewed in the first wave in 2003 (*N* = 1937) with the distributions in the analysed sample interviewed in 2009 (*N* = 1362). In the latter, 24.8 % of the fathers had college or university education, as against 21.4 % in the 2003 sample, and the proportion of non-Western families was reduced from 18.0 % in 2003 to 14.7 % in 2009. Attrition from 2003 to 2009 was therefore more marked among certain family categories, but the 2003 and 2009 samples had nearly the same proportions of intact families and of parents with “very good” self-rated health. In conclusion, it is not probable that sample bias has led to drastically distorted results, in particular because attrition has resulted in an “upwardly biased” sample where it is likely that associations with socioeconomic indicators could have been attenuated.

### Implications for Child and Adolescent Indicators?

The results could be relevant for how indicators of a family’s economic situation should be constructed in childhood research. Family income could be a one-sided measurement; it is striking that family income had rather small associations with perceived parenting in this study, compared to the associations which emerged when the total family economy indicator was utilised. Rising affluence in rich countries could have implied that many families’ material standard of living, as well as their feelings of economic security, have become more linked to wealth and less reliant on current income. This suggests that financial assets or other indicators for family wealth should be taken into account when the economic resources in the families of children and adolescents are measured.

Moreover, this study implies that indicators of parenting could be useful child indicators. As argued in the Introduction, it seems documented that the quality of parenting is associated with an array of outcomes representing both the well-being and the well-becoming aspects of children’s and adolescents’ lives. Adolescents’ assessment of the parent-offspring relation is relevant not only because of the associations between parenting and other outcomes of interest, but in its own right: Viewing parents as responsive, interested, but not uncomfortably intrusive, will constitute an important aspect of a satisfying parent-offspring relationship as seen by the adolescent.

That parenting-related aspects could be included in systems monitoring child well-being, has indeed been recognised in recent child indicators research. “From the earliest years, the child’s sense of subjective well-being is intimately bound up with relationships, and particularly with parents and peers”, was pointed out in a recent UNICEF report (UNICEF 2013:40). A study based on the UNICEF project noted that family relationship is “the single most important contributor to children’s subjective well-being” (Bradshaw et al. 2013: 623). In the UNICEF project, an index of children’s subjective well-being was defined in terms of life satisfaction, relationships, school experiences, and health, with data from an international survey among school children. Two of the three questions used to measure relationship quality were related to parenting, as the children were asked whether they found it “easy to talk to their mother” and “to their father” (Bradshaw et al. 2013). These questions correspond well to two of the items used by the present study for constructing the responsiveness parenting dimension: “I tell my parents about my problems” and “My parents understand me” (see Appendix Table 4). The two items used in the UNICEF study will not capture the nuances of the parenting style concept and the varieties in adolescents’ views on parenting, but they may nevertheless be good approximations with considerable informational value. It can be noted that Norwegian children scored around the middle among the 29 rich countries analysed in the UNICEF study as regards the “easy to talk to mothers/fathers” items (Bradshaw et al. 2013:625). The present study adds to this information by showing social variations around this national average.

A difficult issue is how to decide cut-off points which distinguish meaningfully between clearly satisfying and unsatisfactory parenting. In the UNICEF study, response alternatives ranged from “very easy” to “very difficult”, and the percentages answering “very easy” and “easy” in each country were reported. Whether this cut-off level reflects adequately a positive experience of parenting is open to further research. In the present study, the same difficulty is present, as we do not know how particular values on the parenting dimension scales are associated with other well-being measures.

## Conclusion

Differences in responsiveness and demandingness, but also variations in neglecting and intrusive parental behaviours, capture large parts of the variations in Norwegian adolescents’ experiences of parenting. Responsiveness is a particularly important dimension. Adolescents in families with fewer economic resources experience their parents as somewhat less responsive and supportive, but responsiveness appears to have no associations with parental education. Low parental education seems on the other hand to be related to more frequent perceptions of parents as neglecting and intrusive. The demandingness dimension, signifying the monitoring and controlling attitudes of the parents, did neither vary with parental education nor with families’ economic resources. Social variations in parenting dimensions in contemporary Norway seem relatively restricted, however, but the link between perceived parental responsiveness and the families’ economic resources is noteworthy.

## Appendix

**Table 4:** Factor analysis, 17 parenting items, principal component analysis, varimax rotation, four extracted factors. Parameters and correlations for the scales

Question \ rotated component matrix	1	2	3	4
1. My parents accept me as I am	0.615	0.053	−0.246	0.004
2. I tell my parents about my problems	0.688	0.309	−0.055	−0.042
3. My parents understand me	0.748	0.201	−0.159	−0.008
4. I do trust my parents	0.601	0.264	−0.294	0.049
5. My parents appreciate that I make own decisions	0.378	−0.018	−0.171	−0.243
6. My parents have understood my problems	0.552	−0.007	−0.011	−0.031
7. Very important to come home on time in evenings	−0.022	0.704	−0.049	0.145
8. Very important to do my homework for school	0.109	0.637	−0.067	0.021
9. Very important to behave well towards other	0.046	0.589	−0.262	−0.079
10. Very important to be helpful at home	0.069	0.534	0.049	−0.159
11. My parents usually know where I am…	0.386	0.546	−0.054	0.039
12. Important for my parents to know where I am	0.273	0.614	−0.091	0.196
13. My parents have not talked much with me	−0.154	−0.083	0.704	0.005
14. My parents have not helped me much	−0.132	−0.067	0.703	0.109
15. My parents have spent too little time with me	−0.222	−0.114	0.701	0.073
16. My parents have always tried to control what I do	0.002	0.076	0.127	0.783
17. My parents have protected me too much	−0.080	−0.026	0.018	0.806
Initial Eigenvalues	4.14	1.81	1.21	1.08
Per cent explained of variation	24.4	10.6	7.1	6.3
Scales	Respon-sive	Deman-ding	Neglec-ting	Intru-sive
Number of answers	1286	1307	1308	1300
Number of items	6	6	3	2
Cronbach’s alpha	0.686	0.685	0.624	0.540
Mean	4.40	4.19	1.50	2.54
Standard deviation	0.50	0.73	0.66	1.12
Correlations (Pearson’s r)				
Factor 1 Responsive	1	0.436	−0.437	−0.104
Factor 2 Demanding		1	−0.262	0.077
Factor 3 Neglecting			1	0.173
Factor 4 Intrusive				1
